# Does Emotional Arousal Influence Swearing Fluency?

**DOI:** 10.1007/s10936-016-9473-8

**Published:** 2017-01-16

**Authors:** Richard Stephens, Amy Zile

**Affiliations:** School of Psychology, Keele University, Keele, Staffordshire, ST5 5BG United Kingdom

**Keywords:** Taboo, Swearing, Verbal fluency, Emotion, State hostility, First person shooter

## Abstract

This study assessed the effect of experimentally manipulated emotional arousal on swearing fluency. We hypothesised that swear word generation would be increased with raised emotional arousal. The emotional arousal of 60 participants was manipulated by having them play a first-person shooter video game or, as a control, a golf video game, in a randomised order. A behavioural measure of swearing fluency based on the Controlled Oral Word Association Test was employed. Successful experimental manipulation was indicated by raised State Hostility Questionnaire scores after playing the shooter game. Swearing fluency was significantly greater after playing the shooter game compared with the golf game. Validity of the swearing fluency task was demonstrated via positive correlations with self-reported swearing fluency and daily swearing frequency. In certain instances swearing may represent a form of emotional expression. This finding will inform debates around the acceptability of using taboo language.

## Introduction

Previously it has been shown that swearing, defined as the use of offensive or in some instances taboo language (Soanes 2002), can increase tolerance for pain (Stephens et al. 2009; Stephens and Umland 2011). Research has found that participants repeating a swear word could hold their hand in ice-cold water for longer than when they repeated a neutral word and that the increased pain tolerance was accompanied by increased heart rate. The apparent mechanism is one where swearing increases a speaker’s emotional arousal leading to a stress-induced analgesia as part of the fight or flight response (Xie et al. 2008).

This is in keeping with the idea that in certain situations swearing can represent a linguistic expression of emotion, evidenced by the findings that swear words are subjectively rated as emotionally arousing (Janschewitz 2008) and that swearing elicits a skin conductance response (Bowers and Pleydell-Pearce 2011; Jay et al. 2008). Functionally, swearing has been linked with the deeper-lying emotion centres of the brain (Van Lancker and Cummings 1999).

While there is evidence that swearing can induce emotion, whether the opposite of this is true—that emotional activation can elicit swearing—is less clear, although anecdotal evidence supports such a link. One example of this was when the athlete Bryony Shaw was captured live on daytime TV spontaneously expressing her euphoria upon gaining an unexpected Olympic Bronze medal, proclaiming “I’m so *fucking* happy” (Telegraph 2008). Assuming that Shaw did not intentionally set out to cause offence by using taboo language, this may be an example of emotional arousal influencing aspects of swearing production such as lexical access and the disinhibition of moderating and self-censoring processes.

Several decades ago Ross (1960) recorded the swearing frequencies of five men and three women on a university arctic expedition, and noted an increased frequency of annoyance swearing under conditions of mild stress. However, it is not possible to infer from this correlational finding whether the emotional arousal arising from mild stress “caused” the increased swearing frequency or, alternatively, whether people that are more prone to swear are also more emotionally labile.

It is important to gain a proper psychological understanding of the link between emotional arousal and swearing on both theoretical and applied fronts. A rapidly growing literature on emotional regulation has theorised that individuals may choose deliberately to exaggerate their emotional responses in a behaviour known as *venting* (Koole 2009). Swearing as a response to emotive episodes may perform emotional regulation functions along these lines, although research assessing direct links between swearing and the experience of emotion is limited. Improving understanding of how emotional arousal may impact on swearing behaviour would also inform public debate on the place of swearing in society. For instance, Section 5 of the UK Public Order Act 1986 makes it an offence to use threatening, abusive or insulting words or behaviour within the hearing or sight of a person likely to be caused harassment, alarm or distress (Strickland and Douse 2013). Given the degree of subjective interpretation possible within this statute, a better understanding of the link between emotional arousal and swearing, and particularly whether the former is likely to lead to the latter, would assist in interpreting Section 5 so that the law can be applied fairly.

The present studies used a swearing fluency task (SFT) based on the Controlled Oral Word Association Test of verbal fluency (COWAT; Ruff et al. 1996) as a measure of swearing production. The SFT requires participants to generate as many swear words as possible in 1 min. In a previous study of swearing fluency, Jay and Jay (2015) compared word generation rates across three different prompts: letters (following the procedure of the COWAT), animal words, and swear words (following the procedure of the SFT). Positive correlations between generation scores to all prompts were found challenging the hypothesis that swearing is a sign of an impoverished vocabulary.

Here we explore, experimentally, how swearing fluency can vary within a speaker. Specifically we examined the relationship between emotional arousal and swearing fluency. In Experiment 1 we carried out some psychometric work further developing the SFT. Construct validity was assessed with reference to self-reported swearing fluency, general swearing frequency and comparisons between men and women. Experiment 2 comprised a within-subjects comparison of the effect of manipulated emotional arousal on swearing fluency. In order to manipulate emotional arousal, participants’ state aggression was raised using the previously validated method of playing a first-person shooter (FPS) video game (Stephens and Allsop 2012). This game required the exploration of a virtual three-dimensional environment while continuously exchanging weapon fire with a variety of hostile characters. The control condition was a golf video game. The State Hostility Questionnaire (Anderson et al. 1995) was employed as a manipulation check to verify differences in emotional arousal across the conditions. Swearing fluency was then assessed via the SFT and additionally using a self-report visual analogue scale. Age, sex, daily swearing frequency and IQ were also assessed. These latter two variables were assessed as covariates because either could influence swearing fluency independently of emotional arousal. In particular, IQ was measured with the findings of Jay and Jay (2015) in mind. Their finding that swearing fluency was correlated with general verbal fluency implies that IQ would correlate positively with swearing fluency, although this has not been assessed.

It was hypothesised that emotional arousal, as indicated by state hostility scores, would be greater after 10 min of playing a FPS video game compared with a golf video game. A further hypothesis was that swearing fluency would be greater in the emotion arousing condition of playing the FPS video game compared with the golf video game. Finally, it was hypothesised that IQ and swearing fluency would be positively correlated.

## Experiment 1: The Swearing Fluency Task

### Aims

Experiment 1 aimed to assess the construct validity of the SFT.

### Participants

The participants were 30 undergraduate and postgraduate students recruited from Keele University, consisting of 17 women and 13 men aged 18–43 years ($M = 21.63$; $SD = 4.61$). The Keele University Research Ethics Review Panel approved the study. The only inducement to participate was the promise of some sweets that were given out at the end.

### Design

The case for construct validity was based on two premises. The first was demonstrating significant positive correlations between scores on the SFT and self-reported swearing fluency and self-reported daily swearing frequency. Therefore, this aspect of Experiment 1 applied a correlational design. The second premise of the case for construct validity was based on demonstrating an absence of sex differences in swearing fluency, mirroring the previously reported absence of sex differences in swearing fluency (Jay and Jay 2015). Therefore this aspect of Experiment 1 applied a between-subjects (men vs. women) comparison of self-reported swearing frequency, self-reported swearing fluency and SFT scores.

### Materials

#### SFT

The SFT was based on the COWAT (Ruff et al. 1996) and required participants to write down as many different swear words as they could think of in 1 min. Instructions were developed in order to ensure that participants spent the allotted time trying to think of different epithets rather than repeating similar variations on one word or expression, as follows: *In this test I would like you to write down as many swear words as you can think of in one minute. Do you know what a swear word is? You may use compound swear words that re-use a word you have already said. So for example, if you had already said ‘fuck’ then you could add ‘fuck-face’. However, in doing this the compound (double-barrelled) swear word must be a recognised linguistic form. So, for example, saying ‘fuck-table’ would not count, as this is not a recognised swear word. ‘Fucking idiot’ would not count, as that is two words. Do you understand?* Participants were provided with an answer booklet for writing down their responses. Participants received one point for each word they produced that was recognised by the authors as a swear word. Duplicate words and expressions not recognised as *bona fide* swearing by the experimenters were excluded from the score. The latter are reported.

#### Self-Reported Swearing Fluency

A visual analogue scale was used to assess self-reported swearing fluency. The scale comprised of a line on a page 100 mm long, with an anchor at each end of the line. Participants were asked, *Please indicate on the line below how fluent you feel you are at swearing, on average*. The anchors were *0% fluent (not at all)* on the left, and *100% fluent (as fluent as possible)* on the right. Participants responded by making a mark on the line that was converted into a percentage score by measuring in mm from the left anchor.

#### Self-Reported Daily Swearing Frequency

Daily swearing frequency was assessed by asking participants to estimate the frequency with which they swear over one of three timescales. Participants were asked: *On average, how often do you swear? Please indicate choosing ONE timescale out of ‘swear words per day’, ‘swear words per week’ or ‘swear words per month’. Choose the timescale that is most appropriate for you*. This was the same method used by Stephens and Umland (2011).

### Procedure

Testing was carried out one-to-one, in private, either in a research lab in the School of Psychology, or in a pre-booked private study room in the library. After completing the consent form, participants were asked their age and their sex was recorded. Next they completed the SFT, the self-reported swearing fluency measure and the self-reported daily swearing frequency measure. Finally, participants were debriefed, provided with the opportunity to ask any questions about the study, and thanked.

### Results

Descriptive data are shown in Table 1. All dependent variables followed a normal distribution except daily swearing frequency, which was right skewed. Applying a log computation did not completely transform this variable to normal, therefore, both transformed and untransformed analyses are reported. On the SFT the number of words generated ranged from 4 to 15. A total number of 37 unique swear words were generated across the entire sample. Only one word, *curse*, was considered not to be a recognised swear word.

**Table 1:** Means (SDs) of participant descriptive data (age, Swearing Fluency Test score, self-reported swearing fluency and daily swearing frequency) by sex

Variables	Males	Females
	$n = 13$	$n = 17$
Age	20.38	22.59
	1.71	5.95
SFT score	8.31	7.18
	3.33	1.98
Self-reported swearing fluency (%)	52.62	46.29
	21.93	26.00
Daily swearing frequency	15.65	8.77
	19.18	4.87

The validity of the SFT was initially assessed by examining correlations between SFT scores, self-reported swearing fluency and self-reported daily swearing frequency. There was a significant correlation between SFT scores and self-reported swearing fluency, $r(30) = 0.578$, $p = 0.001$. There was also a significant correlation between SFT task scores and self-reported daily swearing frequency, untransformed $r(30) = 0.496$, $p = 0.005$; transformed $r(30) = 0.375$, $p =0.041$. Validity of the SFT was further assessed with reference to sex differences. There were no sex differences for SFT scores, $t(28) = 1.163$, $p = 0.255$, $d = 0.255$, for self-reported swearing fluency, $t (28) = 0.705$, $p = .487$, $d = 0.261$, or for self-reported swearing frequency, $t (28) = 1.426$, $p = 0.165$ $d = 0.621$.

### Discussion

Experiment 1 showed that individuals were able to demonstrate their swearing fluency by writing down as many swear words as they could think of in 1 min, as required by the SFT. The mean score of 7.63 (*SD* = 2.68) is comparable with but below the scores obtained in the written form of the SFT employed by Jay and Jay (2015) of 10.88 (*SD* = 4.14) in Study 2, and 11.07 (*SD* = 4.00) in Study 3. Moreover, SFT scores were positively and significantly correlated with individuals’ own estimates of their swearing fluency and with individuals’ own estimates of their daily swearing frequency. These correlations, showing that SFT scores match participants’ opinions of their own swearing fluency, and that more frequent users of swearing and taboo language are more fluent—which is to be expected due to increased familiarity—substantiate the construct validity of the SFT. The consistent absence of sex differences in the SFT, self-reported swearing fluency and self-reported swearing frequency also contributes to the case for validity since all three measures were in agreement with previous findings of absence of sex differences in swearing (Jay and Jay 2015).

## Experiment 2: Emotional Arousal and Swearing Fluency

### Aims

Experiment 2 aimed to assess the effect of emotional arousal on swearing fluency. Emotion was manipulated by having participants play an FPS video game (experimental condition) compared with a golf video game (control condition). It was hypothesised that emotional arousal, as indicated by state hostility scores, would be greater after playing the FPS compared with the golf game. Swearing fluency was measured after each game play session. It was further hypothesised that swearing fluency would be greater following the FPS game due to heightened emotional arousal. Daily swearing frequency and IQ were also measured in order to assess whether these variables moderate or mediate the effect of emotional arousal on swearing fluency.

### Participants

The participants were 60 undergraduate and postgraduate students recruited from Keele University, consisting of 33 women and 27 men, aged 18–43 years ($M = 21.25$; $SD = 3.47$). The Keele University Ethical Review Panel approved the study. The only inducement to participate was the promise of some sweets that were given out at the end.

### Design

A repeated measures design was applied in which State Hostility Questionnaire Score, SFT score and swearing fluency visual analogue scale score were compared after 10 min of playing either a FPS video game, implemented to increase arousal, or a golf video game implemented as a neutral control condition. Condition order was randomised across participants.

### Materials

#### Video Games

The Medal of Honor Frontline FPS video game (Games 2002) was employed with the aim of inducing increased emotional arousal in participants. The Tiger Woods PGA tour 2007 golf video game (Games 2006) was employed as a control condition. Game play and conditions were identical to those reported previously (Stephens and Allsop 2012).

#### State Hostility Questionnaire

Emotional arousal, and specifically aggressive affect, was assessed using the State Hostility Questionnaire (Anderson et al. 1995). The original version of this questionnaire requires participants to rate 35 items (*e*.*g*., “I feel furious”) on a 5-point scale from 1 (“strongly disagree”) to 5 (“strongly agree”). Anderson advises that 3 of the items (“wilful”, “tender” and “vexed”) sometimes yield poor item-total correlations, impacting upon reliability. We required two alternate forms of the State Hostility Questionnaire; one to be applied after each of the video games to assess whether heightened emotional arousal had successfully been induced. Therefore, replicating a previous study in our laboratory, we removed those 3 items and divided the remaining items into two 16-item questionnaire forms, the scores on which could range from 16 to 80. Acceptable levels of reliability (Cronbach’s alpha $>0.9$) for these two forms has previously been demonstrated (Stephens and Allsop 2012).

#### Other Measures

Swearing fluency was assessed using the SFT and the swearing fluency visual analogue scale, both as described in Experiment 1. Self-reported daily swearing frequency was also assessed using the same method as reported for Experiment 1. The National Adult Reading Test (NART; Nelson and Wilson 1991) was used to assess IQ.

### Procedure

Participants individually attended a sound attenuated research laboratory. At the outset participants were informed that they would be taking part in a study about the manner in which swearing can affect people in body and mind. First, the NART was completed. Next, after receiving instruction, participants played one of the video games for 10 min, after which they completed the State Hostility Questionnaire, the SFT, the swearing fluency visual analogue scale and the estimate of daily swearing frequency. This procedure was then repeated with the other video game.

### Results

All variables followed a normal distribution although tending towards leptokurtosis in some cases. However, where appropriate transforms could be identified (*e*.*g*., a logarithmic transform was applied to the State Hostility Questionnaire scores), analyses yielded identical results. Therefore, only non-transformed analyses are reported. Descriptive data are shown in Table 2.

**Table 2:** Means (SDs) of State Hostility Questionnaire score, SFT score and Swearing Fluency VAS score by study condition and sex; and age and covariate scores by sex; *p* values are for male versus female comparisons using unpaired *t* tests

Variables	Males	Females	*p*
	$n = 27$	$n = 33$	
Age	21.63	20.94	0.448
	4.64	2.12	
State Hostility Questionnaire score			
First person shooter game	43.89	46.12	0.478
	13.79	10.42	
Golf game	37.19	32.55	0.064
	12.25	6.38	
SFT score			
First person shooter game	8.44	8.30	0.844
	2.99	2.57	
Golf game	7.59	6.82	0.296
	3.15	2.54	
Swearing Fluency VAS score			
First person shooter game	67.26	65.58	0.744
	18.32	20.83	
Golf game	62.41	64.09	0.774
	23.90	21.30	
Covariates			
NART score	38.26	35.21	0.026
	3.83	5.98	
Daily swearing frequency	32.52	30.06	0.813
	45.28	34.94	

*VAS* Visual Analogue Scale, *NART* National Adult Reading Test

**Fig. 1:**
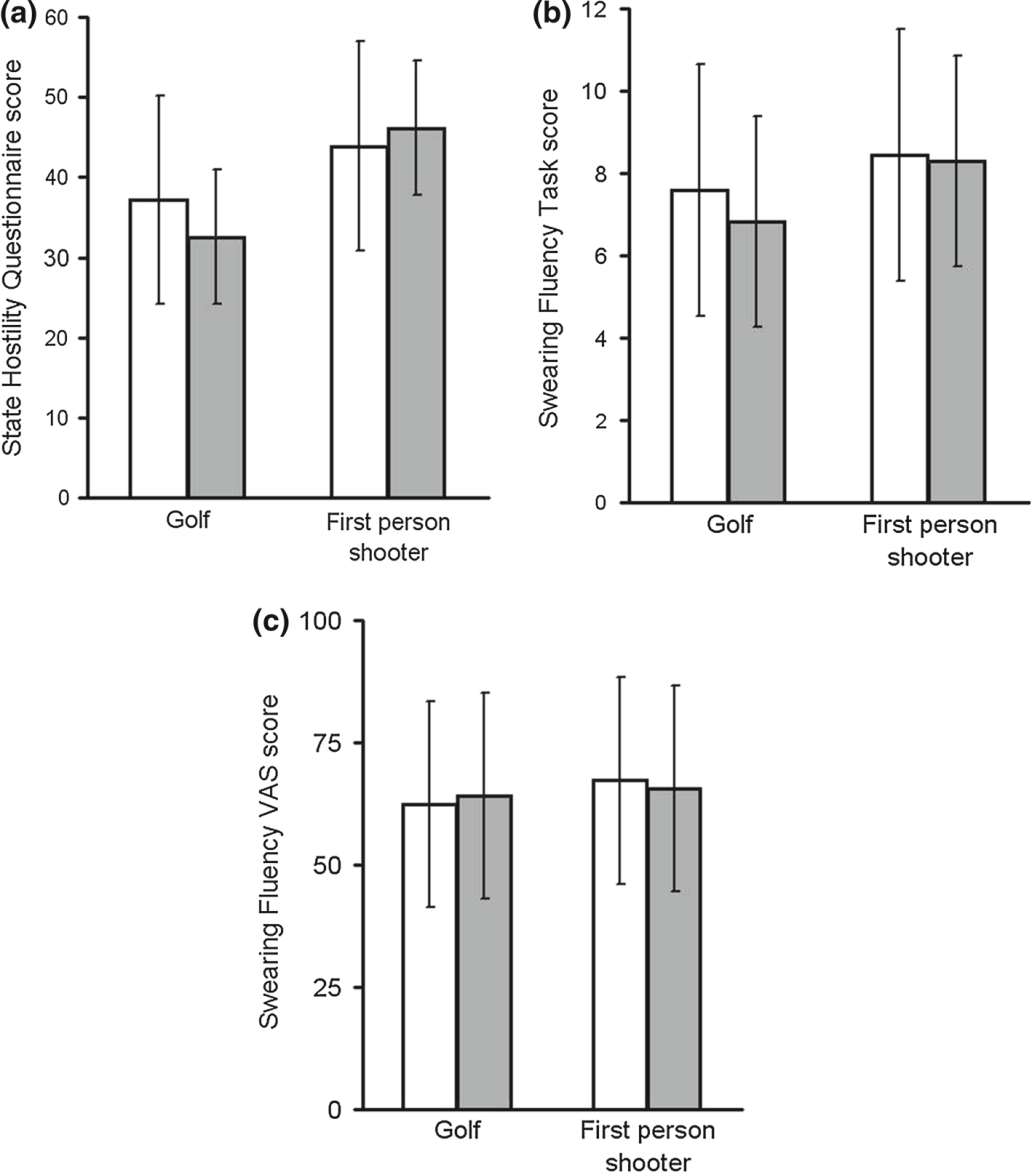
State Hostility Questionnaire score (**a**), SFTscore (**b**) and Swearing Fluency VAS score by game type (golf vs. first person shooter) and sex (males: *white bars*; females: *grey bars*). *Error bars* show the standard deviation

As a check on whether the manipulation was successful, a mixed 2 x 2 ANOVA was used to investigate the effect of 10 min of playing video games (FPS vs. golf) and sex (men vs. women) on State Hostility Scale Questionnaire scores. There was a significant main effect of game type, $F(1, 59) = 65.990$, $p < 0.001$, $\eta ^{2} = 0.532$, such that State Hostility Questionnaire scores were higher after playing the FPS game. This is consistent with raised state aggression levels after playing the FPS game (see Fig. 1a). There was no significant main effect of sex, $F(1,58) < 1.0$, but there was a significant game by sex interaction, $F (1,58) = 7.578$, $p = 0.008$, $\eta ^{2} = 0.116$. The interaction reflected a larger increase in State Hostility Questionnaire scores from the golf game to the FPS game in women.

For the SFT scores, 37 participants (62% of the sample) had an increased score after playing the shooter game compared with after playing the golf game, 14 participants (23% of the sample) had the same score and 9 participants (15% of the sample) had a lower score. This distribution of frequencies is not what would be expected by chance, chi-square = 22.300, *df* = 2, $p < 0.001$, $w = 0.62$. Overall 60 different swear words were recorded. Nineteen words were deemed not to be a recognised linguistic form of swear word. These were: *asstaxi*; *bastarding*; *bitchtwat*; *cuntbag*; *cuntbombination*; *cuntbucket*; *cuntsuck*; *dickhole*; *feck*; *fuckeroo*; *fucknose*; *fucktoy*; *penis*; *shitcast*; *suckfucker*; *thundercunt*; *twatbag*; *twathat*; *wanko*.

Mixed 2 x 2 ANOVAs were used to assess the effect of game and sex on SFT score and swearing fluency visual analogue scale score. Following a similar pattern to that observed for the manipulation check data, there were significant main effects of game on SFT score, $F (1,58) = 18.688$, $p < 0.001$, $\eta ^{2} = 0.244$, and on swearing fluency visual analogue scale score, $F (1,58) = 4.702$, $p = 0.034$, $\eta ^{2} = 0.075$. Both measures of swearing fluency were raised following the FPS game (see Fig. 1b, c). There were no significant effects of sex and no significant interaction effects.

Separate and simultaneous general linear model (GLM) analyses were applied to each of the dependent variables: SFT score and swearing fluency visual analogue scale score. Each analysis included the qualitative predictors – game and sex – as well as one of the following centred (Cohen et al. 2003) quantitative predictors: NART score; or daily swearing frequency. In each analysis, to check regression homogeneity, first the 3-way interaction was examined in a GLM additionally containing all of the 2-way interactions and the main effects. If the 3-way interaction was not significant, then a GLM including only the 2-way interactions and the main effects was inspected. Where none of the interactions was significant, a final GLM including only the main effects, equivalent to traditional analysis of covariance was applied. Prior to conducting the GLM analyses, the correlation between NART score and daily swearing frequency was calculated, but was not significant, $r = 0.247$, $p = 0.057$.

NART score did not predict SFT score as part of a three-way interaction with game and sex, $F (1, 56) = 1.146$, $p =0.249$, $\eta ^{2} = 0.020$, or in a GLM in which the three-way interaction was not included, either as part of an interaction with game, $F (1,57) = 1.103$, $p = 0.298$, $\eta ^{2} = 0.019$, or with sex, $F(1, 56) < 1.0$, or as a main effect, $F(1, 57) < 1.0$. Daily swearing frequency did not predict SFT score as part of a three-way interaction with game and sex, $F (1, 56) < 1.0$, or in a GLM in which the three-way interaction was not included, either as part of an interaction with game, $F (1, 57) < 1.0$, or with sex, $F(1, 56) < 1.0$, or as a main effect, $F(1, 57) = 1.214$, $p = 0.275$, $\eta ^{2} = 0.021$.

To check that condition order effects did not unduly influence the above results, these were examined via a series of 2 x 2 mixed ANOVAs for the dependent variables: State Hostility Questionnaire score, SFT score and swearing fluency visual analogue scale score. Each ANOVA included the between-subjects factor condition order (golf first vs. FPS first) the within-subjects factor game (golf vs. FPS), and the game x condition order interaction. Table 3 summarizes the means and standard deviations examined in these analyses.

**Table 3:** Means (SDs) of State Hostility Questionnaire score, SFT score and Swearing Fluency VAS score by condition order

Variables	Golf first	Shooter first
	$n = 27$	$n = 33$
State Hostility Questionnaire score		
First person shooter game	46.77	43.47
	11.28	12.65
Golf game	34.93	34.33
	8.22	11.08
SFT score		
First person shooter game	8.57	8.17
	2.01	3.34
Golf game	7.13	7.20
	2.46	3.21
Swearing Fluency VAS score		
First person shooter game	68.23	64.43
	21.32	17.86
Golf game	63.33	63.33
	24.69	20.12

*VAS* Visual Analogue Scale

Condition order did not predict State Hostility Questionnaire score, SFT score or swearing fluency visual analogue scale score, either as part of a game x condition order interaction, $F (1, 58) < 1.720$, $p> 0.195$, $\eta ^{2 }< 0.029$, or as a main effect, $F (1, 58) < 1.0$.

Finally, to assess the reliability of the SFT, the correlation of participants’ scores across the FPS game and golf game conditions was calculated, $r = 0.720$, $p < 0.001$.

### Discussion

Experiment 2 assessed, experimentally, the relationship between emotional arousal and swearing fluency. Playing the FPS video game was found to increase emotional arousal (in terms of state hostility score) compared with a golf video game. This supports our first hypothesis. Swearing fluency also increased when participants were in a heightened state of emotional arousal. This supports our second hypothesis that swearing fluency would be increased due to emotional arousal. However, there was no association between swearing fluency, measured using the SFT, and intelligence, measured using the NART, or between swearing fluency, measured using the SFT, and self-reported daily swearing frequency. Experiment 2 also demonstrated that the SFT has good reliability since the observed test-retest correlation ($r = 0.720$) was very similar to the test-retest correlation of the COWAT ($r = 0.74$; Ruff et al. 1996).

## General Discussion

Here we present the first study to investigate the effect of laboratory-induced emotional arousal on swearing fluency. Our hypothesis that swearing should come more naturally when individuals experience higher than usual levels of emotional arousal was supported. A debate on how emotion is rendered neurologically, and particularly how emotional and cognitive processes are integrated has been ongoing since Schachter and Singer (1962) proposed the two-factor theory of emotion. These debates are relevant to the present study in which increased swearing fluency can be viewed as a confluence of manipulated emotional arousal and the consequent activation of cognitive processes supporting language production. Recent formulations of the two-factor theory of emotion posit that the experience of emotion is predominantly rendered through working memory via the prefrontal cortex with input from the phylogenically older sub-cortical emotion centres of the brain (LeDoux 2000).

Sub-cortical brain regions have also been linked with swearing and taboo language. In their comprehensive review of case studies of aphasia patients and studies of Gilles de la Tourette’s syndrome patients, Van Lancker and Cummings (1999) argue that some forms of swearing represent automatic speech that is less reliant on the left-hemisphere cortical regions usually associated with speech and language. They cite several studies indicating that, compared with controls, the basal ganglia of Tourette’s patients showed reduced volume, higher glucose activity, diminished blood perfusion and increased dopamine receptor binding. Van Lancker and Cummings suggested that the basal ganglia form a likely origin for swearing linked with activity in the limbic system. They theorized that coprolalia (i.e. the Tourette’s swearing tic) was a type of limbic vocalization associated with a social communicative function (e.g. repulsing intruders or expressing anger) and an exemplar of a phylogenetically older speech system. It follows from this analysis that emotional arousal might lead to increased swearing fluency due to activating of sub-cortical brain regions that facilitate both emotional arousal and taboo language production.

Several possible mechanisms further explain how raised emotional arousal may facilitate swearing fluency. Gawda and Szepietowska (2013) showed in a correlational study that natural variations in emotional arousal can predict verbal fluency. Their cross-sectional study showed that the degree of arousal of participants’ positive affective state was correlated with verbal fluency for positively valenced emotional words. However, there was no corresponding effect for arousal of negative affective state correlating with fluency for negatively valenced emotional words. These authors conjectured that the degree of arousal of positive affective state may lead to increased verbal fluency via an increase in the number of available associations, an increase in cognitive flexibility or a defocussing of attention via unconscious emotional schemata.

Support has also been found for the situational model theory in explaining how mental representations of a situation can transform verbal production (Kaup 2001). The situational model theory posits that we understand discourse by building mental representations such that language can be viewed as a set of processing instructions enabling the individual to build a mental representation of a described situation (Zwann and Radvansky 1998). It follows from this, conversely, that activating mental representations, and specifically ones with emotional connotations, is likely to influence aspects of language production such as lexical access and/or the disinhibition of self-censoring processes. Thus the situational model theory may also explain how emotional arousal may influence verbal production. Further research would be required to understand which of these different processes combine to produce increased swearing fluency.

Previously it has been theorised that swearing can increase pain tolerance via the mechanism of increased emotional arousal producing a stress-induced analgesia as part of the fight or flight response (Stephens et al. 2009; Stephens and Umland 2011). The present study findings of increased swearing fluency under conditions of raised emotional arousal can be conceptualised as the other side of the coin of the relationship between emotion and swearing. Whereas the previous research showed that swearing can increase emotional arousal, here we have shown that increasing emotional arousal can facilitate swearing—or at least one aspect of it: swearing fluency. This study also provides further support for the thesis that swearing can be a form of emotional language—that one common purpose of swearing is the urgent expression of strongly felt emotion (Jay 2009).

These findings provide support for recent changes in the legal status of swearing in the UK. We have shown that swearing is closely linked with a heightened state of emotional arousal. This implies that swearing is not necessarily employed with the intent to cause harm or distress to another person and in many cases may be a form of emotional expression. In the UK, a 2011 High Court ruled that police officers were unlikely to feel harassed or distressed when members of the public swear. The ruling centred on a case in which a young man swore in earshot of the police carrying out a drugs related stop and search (Wardrop 2011). The ruling was based on the high frequency with which four-letter words are used and heard in everyday life. In this case, the young man did not swear directly at the police, rather, the way he swore is likely to have been a reflection of his own frustration (*e*.*g*., “____ *this man. I ain’t been smoking nothing”*). The present findings contribute to debates around the acceptability of taboo language by showing a relationship between swearing and heightened arousal. Furthermore, swearing in the context of strongly emotive episodes may perform an emotion regulation function similar to venting, as described by Koole (2009). We invite future research to investigate links between swearing and emotional regulation.

## Conclusion

This paper presents evidence that swearing fluency can be reliably and validly assessed and demonstrates that swearing fluency increases when emotional arousal increases. We interpret this finding as indicating that swearing comes more naturally with heightened emotional arousal, and therefore these findings support the notion that, in certain instances, swearing represents a form of emotional expression. This finding will inform debates around the acceptability of using taboo language.
